# UPF1-dependent glycolysis driven by LINC00887 facilitates macrophage M2 polarization in clear cell renal cell carcinoma

**DOI:** 10.3389/fimmu.2026.1833383

**Published:** 2026-09-23

**Authors:** Jiayue Wu, Jiawen Wang, Jingyu Weng, Rongcheng Lin, Wanghai Cai, Jiajing Liao, Liefu Ye, Youcheng Lin, Jinfeng Wu

**Affiliations:** 1 Shengli Clinical Medical College of Fujian Medical University, Department of Urology, Fujian Provincial Hospital, Fuzhou, Fujian, China; 2 Fuzhou University Affiliated Provincial Hospital, Department of Urology, Fujian Provincial Hospital, Fuzhou, Fujian, China

**Keywords:** aerobic glycolysis, CD8+ T cells, clear cell renal carcinoma, LINC00887/UPF1, macrophages

## Abstract

**Introduction:**

LINC00887 plays critical roles in tumor biology, from oncogenesis to metastasis and drug resistance, while the specific mechanism in ccRCC remains unclear. We analyzed the expression profiles of LINC00887 and UPF1 and assessed their correlation with glycolytic activity and M2 macrophage infiltration.

**Methods:**

Here, we performed scRNA-seq to identify distinct UPF1 expression profiles and found significant pathways. A suite of assay kits was utilized to evaluate glycolytic metabolism. Glycolytic gene expression at the mRNA and protein levels was quantified via qRT-PCR and immunoblot. Macrophage polarization and CD8^+^ T cells were analyzed via flow cytometry, and CD8^+^ T cell cytotoxicity was quantified with an LDH release assay. Orthotopic tumor models were built for validation.

**Results:**

We found that highly expressed LINC00887 induced M2 macrophage polarization by activating aerobic glycolysis and promoting lactate buildup. Mechanistically, suppression of UPF1 expression promoted malignant progression of ccRCC. Additionally, UPF1 was found to be negatively correlated with aerobic glycolysis, repressing glycolytic activity and M2 macrophage polarization while bolstering the tumor-killing potential of CD8^+^ T cells.

**Conclusion:**

In summary, LINC00887, through the inhibition of UPF1, escalates glycolytic activity in ccRCC and induces M2 macrophage polarization, impairing the effectiveness of CD8^+^ T cells in combating the tumor.

## 1 Introduction

Based on the latest global cancer epidemiological statistics, renal cell carcinoma (RCC) accounts for over 434, 000 newly diagnosed cases and approximately 156, 000 mortality cases annually, ranking among the most prevalent malignant tumors of the urinary system worldwide (1, 2). As the predominant histological subtype of RCC, clear cell renal cell carcinoma (ccRCC) constitutes 75~80% of all RCC diagnoses (3). Although surgical intervention or radiotherapy can achieve therapeutic relief for early-stage ccRCC patients, metastatic progression or tumor recurrence occurs in nearly one-third of these individuals, which markedly compromises their prognostic outcomes (4). The landscape of ccRCC treatment has been revolutionized by advances in molecular biology and genomics, with immunotherapy as a frontline therapy. Immunotherapy leverages the immune system to target cancer cells (5). Yet, despite improved response rates, resistance mechanisms, either inherent or acquired, can lead to tumor evasion of immune surveillance in some patients, contributing to treatment resistance and disease worsening (6).

Enhanced aerobic glycolysis is particularly pronounced in ccRCC (7, 8), characterized by elevated glycolytic flux, suppressed oxidative phosphorylation, and facilitated formation of an immunosuppressive tumor microenvironment (9). Tumor-associated macrophages (TAM) are key immune cells in the tumor microenvironment and participate in the process of cancer growth, immune regulation, angiogenesis, drug resistance, and metastasis (10). According to their differentiation and activity, TAM are divided into M1 and M2 types (11). Lactic acid produced by glycolysis can affect the transformation of macrophages to M2 type (pro-tumor phenotype) through multiple mechanisms (8). Colorectal cancer generates lactate that drives macrophage polarization towards the M2 phenotype (12). In TNBC, lactic acid activates PI3K-AKT-mTOR through GPR81 and promotes M2 polarization in macrophages (13). Therefore, targeting the signals that alter macrophage polarization in the TME might offer a way to optimize therapy efficacy and rein in cancer aggressiveness.

LncRNAs do not code for proteins but they are not silent in the TME (14), serving as either oncogenes or tumor suppressors to modulate tumor progression, metabolic reprogramming and immune regulation (15). For example, LncRNA MILIP drives ccRCC metastasis by binding YBX1 to translational activation of Snai1 (16). LINC01094 triggers the transformation of glucose metabolism from mitochondrial respiration to glycolysis, thereby promoting the progression of breast cancer (17). Here, we revealed that LINC00887 was significantly upregulated in ccRCC, which facilitated glycolytic metabolism and induced M2 macrophage polarization via negatively modulating UPF1 expression. These findings expand our mechanistic understanding that reduced UPF1 expression can reprogram glycolytic metabolism, leading to an enhanced immunosuppressive tumor microenvironment.

## 2 Materials and methods

### 2.1 Patient samples

As part of our research on ccRCC, we included two matched pairs of tumor and normal tissue samples from the Ethics Committee of Fujian Provincial Hospital (No. K2025-06-010). All subjects gave written consent before participating in the investigation. Every methodology followed established protocols and complied with relevant regulations.

### 2.2 scRNA-seq analysis

Firstly, tissue samples were rinsed with PBS. Excess nontarget tissue was dissected away, and the remaining samples were minced into 1–2 mm fragments before digestion with tissue dissociation buffer (Genergy Bio, China) for 45 min. The digested cell suspension was serially filtered through 70 μm and 40 μm cell strainers, after which low-speed centrifugation was performed to pellet cells. Red blood cell lysis buffer (Solarbio, China) was subsequently added to eliminate erythrocytes. Following cell concentration adjustment, single-cell capture and library preparation were initiated. Single-cell RNA sequencing (scRNA-seq) was conducted using the 10× Chromium Single Cell 3’ Reagent Kit (V3), strictly following the manufacturer’s instructions. Briefly, the single-cell suspension was resuspended and quantified to achieve a target yield of 10, 000 cells per specimen. Libraries were generated via reverse transcription followed by 12 cycles of PCR amplification. Paired-end 150 bp (PE 150 bp) sequencing was run on the Illumina NovaSeq 6000 platform (Illumina, USA), with each sample generating approximately 100 Gb of raw sequencing data. Metabolic activity across individual cells was quantified using the VISION algorithm integrated within the scMetabolism package, in conjunction with curated KEGG metabolic gene sets. For downstream bioinformatic processing, the Seurat package was utilized: the NormalizeData function normalized raw expression matrices, while FindVariableFeatures identified highly variable genes. To mitigate inter-sample batch effects, dataset integration was executed via Seurat’s IntegrateData function. Canonical correlation analysis (CCA), implemented through FindIntegrationAnchors, detected integration anchors to guarantee consistent and comparable cell clustering across distinct samples. With default parameter settings, FindClusters segregated all captured cells into 16 unique clusters, and the UMAP algorithm was applied to visualize cluster distribution. The FindAllMarkers function further identified cluster-specific differentially expressed genes. Cell subtypes were annotated according to well-established canonical marker genes cited in prior published studies, and functional enrichment analyses were performed against KEGG metabolic pathway gene sets. Lastly, CellChat v2 (v2.2.0) was used to separately reconstruct cell–cell communication networks for tumor and adjacent normal tissues. Marker genes applied for cell-type annotation are listed below: Fibroblasts: COL1A1, DCN, FGF7; B cells: CD79A, MS4A1, CD24; Endothelial cells: CLDN5, CDH5, PECAM1, VWF; Epithelial cells: EPCAM, KRT18, KRT19, CD24; T/NK cells: CD3D, CD8A, GZMK, KLRD1, GNLY; Mast cells: TPSAB1, TPSB2; Monocytes/Macrophages: CD14, CD68, ITGAM.

### 2.3 Bioinformatics analysis

From TCGA (https://portal.gdc.cancer.gov/), TCGA-KIRC datasets were downloaded (ccRCC: 501; normal: 69) for Pearson correlation analysis. Wilcoxon tests were applied to analyze the mRNA levels of LINC00887 and UPF1 in both ccRCC and normal tissues. KEGG was used to analyze signaling pathways enriched by LINC00887 in ccRCC. Based on the TCGA-KIRC dataset, the expression levels of UPF1 were analyzed across different ISUP grades, TNM stages and pathological stages. Furthermore, patients were stratified into high-expression and low-expression groups according to the median expression value of UPF1. Kaplan–Meier survival curves were plotted to evaluate overall survival (OS) and progression-free survival (PFS). Pearson’s correlation was used to evaluate the correlation between LINC00887 and glycolysis-associated genes, immune cells, and UPF1. Correlation between LINC00887 and M2 macrophages was calculated using the CIBERSORT-ABS algorithm (18).

### 2.4 Cell culture

We sourced human renal proximal tubular epithelial cells HK-2 (BNCC339833), ccRCC cells including Caki-1 (BNCC100682), 786-O (BNCC338472), and A498 (BNCC100609), as well as the mouse RCC cell line Renca (BNCC337841), human monocytes (BNCC358410), and culture media from BNCC (China). Primary CD8^+^ Cytotoxic T Cells (PCS-800-017) were from ATCC (USA). HK-2, THP-1, and Renca cells were grown in RPMI-1640 (11875093, Gibco, USA). THP-1 cells were given an extra 0.05 mM β-mercaptoethanol (21985023, Gibco, USA), and Renca cells were supplemented with additional non-essential amino acids (PB180424, Procell, China). Caki-1 cells were kept in 5A medium (30-2007, ATCC, USA), 786-O cells in DMEM-H (11965092, Gibco, USA), and A498 cells in EMEM (30-2003, ATCC, USA). All media received a 10% fetal bovine serum (FBS, A5256701, Gibco, USA) and 1% penicillin-streptomycin (15140122, Gibco, USA) supplement. RPMI-1640 medium was used for the cultivation of CD8^+^ T cells, which were activated using Dynabeads™ CD3/CD28 supplied by ThermoFisher (11131D, USA). Differentiation of THP-1 cells into M0 macrophages was induced by a 24-h treatment with 100 ng/mL PMA (HY-18739, MCE, USA). Cells were incubated at 37 °C with a 5% CO_2_ supply, with the incubator also from ThermoFisher (USA).

### 2.5 Construction of plasmids for lentiviral vectors

Full-length sequences of LINC00887 and UPF1 were cloned into the pcDNA 3.1 vector (V79020, ThermoFisher, USA) to generate overexpression vectors oe-LINC00887 and oe-UPF1. shRNA targeting LINC00887 and UPF1, designed by GenePharma (China), were inserted into the pGPU6/Neo vector (C02003, GenePharma, China) to create sh-LINC00887 and sh-UPF1. Transfection of recombinant plasmids and negative controls was performed in target cells using Lipofectamine 2000 (11668019, Invitrogen, USA), with qRT-PCR validation applied to confirm transfection efficiency.

For stable LINC00887 and UPF1 knockdown, we inserted the shRNA sequences into the pLV-Neo lentiviral vector (VL3003, Inovogen Tech. Co., China). Lentiviral particles were obtained by co-transfecting HEK293T cells with vector and packaging plasmids. Stable transduction of Renca cells was conducted at an optimized multiplicity of infection (MOI). Cells were then subjected to antibiotic screening, and qRT-PCR analysis was implemented to verify transduction efficiency.

### 2.6 qRT-PCR

In accordance with the guidelines provided, total RNA extraction from cells and tumor tissues was performed utilizing Trizol reagent from Invitrogen (15596026CN, USA). Reverse transcription for cDNA synthesis was carried out using the PrimeScript™ RT reagent Kit with gDNA Eraser (RR047A, Takara, Japan). Subsequent qPCR amplification was performed with SYBR Green PCR Master Mix (4309155, ThermoFisher, USA) on a CFX96 Touch Real-Time PCR Detection System (Bio-Rad, USA). β-Actin served as the internal reference gene, and gene expression levels were normalized using the 2^-ΔΔCt^ method. Primers are detailed in **Table 1**.

**Table 1:** qRT-PCR primers.

Gene	Primer sequences (5’→3’)
LINC00887	F: TGGCCAGTGTTTCACCTGTT R: TGATTTCCTCCAACGTGCCA
LDH-A	F: CGTCAGCAAGAGGGAGAAAG R: GCCACGTAGGTCAAGATATCC
HK2	F: GGGACAATGGATGCCTAGATG R: GTTACGGACAATCTCACCCAG
GLUT1	F: TCATCGTGGCTGAACTCTTC R: GATGAAGACGTAGGGACCAC
TNF-α	F: ACTTTGGAGTGATCGGCC R: GCTTGAGGGTTTGCTACAAC
iNOS	F: GTTTGACCAGAGGACCCAG R: ATCTCCTTTGTTACCGCTTCC
IL-1β	F: ATGCACCTGTACGATCACTG R: ACAAAGGACATGGAGAACACC
Arg1	F: CGCCAAGTCCAGAACCATAG R: TCCCCATAATCCTTCACATCAC
Fizz1	F: ACAGTCTAGTAGTACAGTCCCTC R: AACATCCCACGAACCACAG
IL-10	F: TGCCTAACATGCTTCGAGATC R: GGGTCTTGGTTCTCAGCTTG
UPF1	F: CGGAAGCTGGTCAACACTATC R: ATGGGTCTGGAAGTACATGC
β-actin	F: ACCTTCTACAATGAGCTGCG R: CCTGGATAGCAACGTACATGG

### 2.7 Immunoblotting

Total protein was isolated from cells and tumor tissues using RNA immunoprecipitation (RIP) lysis buffer (P0013B, Beyotime, China). Protein content was assessed using a BCA kit (P0012, Beyotime, China). Equivalent protein loads separated by SDS-PAGE followed by PVDF membrane transfer. After blocking with Western blocking buffer (P0023B, Beyotime, China) for 10 min, membranes were incubated at 4 °C with various primary antibodies overnight. On the subsequent day, primary antibodies were removed through washing, after which membranes were exposed to secondary antibodies for 60 min. After additional washes, proteins were detected using a super-sensitive ECL detection kit (P0018FS, Beyotime, China) and visualized on a ChemiDoc imaging system (BIO-RAD, USA). All antibodies used in this study were purchased from Abcam (UK), including rabbit antibodies against Lactate Dehydrogenase A (LDH-A) (ab52488), Hexokinase 2 (HK2) (ab209847), Glucose Transporter 1 (GLUT1) (ab115730), UPF1 (ab109363), β-actin (ab5694), and a goat anti-rabbit IgG secondary (ab6721).

### 2.8 ECAR and OCR

Seahorse XF Pro Analyzer (Agilent) was used to determine ECAR and OCR using the Seahorse XF Glycolysis Stress Test (103020-100) and Mito Stress Test (103015-100) kits, respectively. Briefly, 10, 000 cells were seeded per well in a Seahorse XF 96-well plate (103794-100). Sequential injection of glucose, oligomycin, and 2-DG was performed for ECAR measurement, whereas oligomycin, FCCP, antimycin A, and rotenone were sequentially administered for OCR detection.

### 2.9 Determination of glucose, malondialdehyde, and ATP levels

We measured the glucose consumption rate using a glucose assay kit (BC2500, Solarbio, China). A total of 5 million cells were resuspended in 1 mL of distilled water and subjected to ultrasonic disruption. The mixture was then boiled for 10 min in a water bath and centrifuged at 8000g for 10 min at 25 °C. Supernatant was harvested for further analysis. In a 1.5 mL EP tube, the sample, reagent one, and distilled water were added sequentially, vortexed, and incubated at 37 °C for 15 min. Absorbance was measured at 505 nm using a microplate reader (ThermoFisher, USA).

Lactic acid content was determined using an L-lactic acid assay kit (BC2230, Solarbio). Briefly, cells or tissues were homogenized, and the resulting supernatant was collected and incubated with detection reagents at 37 °C for 20 min under light-shielded conditions. The mixture was then centrifuged at 10, 000 rpm for 10 min at 25 °C. After discarding the supernatant, the precipitate was resuspended. The absorbance value at 570 nm was measured using a microplate reader.

ATP levels in cells were measured using an ATP Assay Kit from Abcam (ab83355, UK). Cells were lysed with pre-chilled ATP assay buffer, and the supernatant was analyzed. Samples were mixed with the assay reagents and incubated at 25 °C for 30 min. After the addition of color-developing reagent, samples were incubated at 25 °C for another 30 min, and absorbance was recorded at 650 nm.

### 2.10 Flow cytometry

To evaluate cell apoptosis, Annexin V-FITC/PI Apoptosis Detection Kit (E-CK-A211, Elabscience, China) was utilized. Treated cells were collected and resuspended at a density of 1×10^6^ cells/mL. After 10 min of dark incubation with Annexin V−FITC and PI staining solutions, cell apoptosis was analyzed using a flow cytometer (ThermoFisher, USA).

Using flow cytometry, we aimed to evaluate polarization levels of human macrophages and ratios of macrophages and CD8^+^ T cells in mouse tumor tissues. Cells or tissues were prepared into single-cell suspensions (1×10^6^ cells/mL) and incubated with respective antibodies for 30 min at 4 °C in the dark. After washing off unbound antibodies, cellular fluorescence intensity was quantified using the flow cytometer. Antibodies included: FITC Mouse monoclonal to CD68 (ab134351, abcam, UK), Pacific Blue™ anti-human CD86 Antibody (305417, yajimall, China), CD206 (MMR) Monoclonal Antibody (19.2)-PE (12-2069-42, ThermoFisher, USA), PerCP/Cyanine5.5 Anti-Mouse F4/80 Antibody (E-AB-F0995J, Elabscience, China), PE Anti-CD206 mouse antibody (E-AB-F1135D, Elabscience, China), APC Anti-mouse CD3 antibody (E-AB-F1013E, Elabscience, China), PE Anti-mouse CD8 alpha antibody (E-AB-F1104D, Elabscience, China).

The Carboxifluorescein Diacetate Succinimidyl Ester (CFSE) Proliferation Kit (C34554, ThermoFisher, USA) was employed to assess CD8^+^ T cell proliferation. CD8^+^ T cells were resuspended in CFSE staining solution and maintained for 20 min at 37 °C. Following washing to remove unbound dye, cells were pelleted and resuspended in pre-warmed Optimem T Cell Expansion Serum-Free Medium (51985034, ThermoFisher, USA). After activation with Dynabeads™ Human T Activator CD3/CD28 (11131D, ThermoFisher, USA), the CD8^+^ T cells were co-cultured, and their fluorescence intensity was examined.

### 2.11 Determination of cell viability

Cell viability of ccRCC cells subjected to different treatments was assessed using the CCK−8 assay kit (C0037, Beyotime, China). Briefly, 2, 000 cells were seeded into each well of a 96−well plate and cultured for 0, 24, 48, 72 and 96 h. At each indicated time point, 10 μL of CCK−8 working solution was added to every well, followed by an additional 4 h incubation. After thorough mixing, absorbance readings were taken at 450 nm.

### 2.12 Detection of cell migration and invasion

To explore ccRCC cell invasion, 24-well Transwell filters from Corning (3422, USA) were employed. The upper chamber was pre-coated with 100 μL of Matrigel (356234, Corning, USA). We placed 10, 000 treated ccRCC cells in the upper chamber with FBS-free medium, with 20% FBS-enriched medium in the lower chamber. Following 24 h, non-invading cells were removed from the membrane, which was then fixed with 75% alcohol and stained with 0.5% crystal violet (C0121, Beyotime, China). Stained cells were analyzed using an Olympus microscope (Japan).

We assessed migration by wound-healing assay. The treated cells were seeded into 24-well plates (3526, Corning) and cultured until reaching approximately 90% confluence. A straight scratch was made using a sterile pipette tip, followed by medium replacement with serum-free medium for another 24 h of incubation. The wound healing status was microscopically observed (Olympus, Japan) at 0 h and 24 h.

### 2.13 Detection of cell proliferation

The capacity of ccRCC cells to form colonies was tested as a measure of their proliferative potential. Post-treatment, 400 ccRCC cells were cultivated in a 12-well plate (3513, Corning, USA) for two weeks. They were fixed with 75% ethanol and stained with a 0.5% crystal violet solution (C0121, Beyotime, China). Colonies were then photographed and counted.

### 2.14 RIP

RIP assays were executed with the Magna RIP Kit from Millipore (17-700, USA). Cells treated under varying conditions were lysed and incubated with magnetic beads coupled to either an anti-UPF1 antibody (9435S, Cell Signaling) or a nonspecific IgG antibody (12-370, Millipore, USA) overnight at 4 °C. After treatment with proteinase K the next day, qRT-PCR was utilized to quantify LINC00887 levels in the isolated RNA.

### 2.15 Detection of CD8^+^ T cell cytotoxicity

To test CD8^+^ T cytotoxicity against ccRCC cells following various treatments, we used an LDH cytotoxicity assay kit (C0016, Beyotime, China). The procedure began with the addition of10 μL LDH substrate to each treatment group, followed by a 15−min incubation at room temperature away from light, and subsequent supplementation with 100 μL LDH detection reagent. After the reaction was fully underway at room temperature in the dark, absorbance was read at 490 nm.

### 2.16 Animal experiment

The animal assays conducted herein adhered to the protocols of the Fuzhou University Affiliated Provincial Hospital Animal Care and Use Committee (IACUC-FPH-SL-20240220[0076]) and adhered to the ARRIVE guidelines. The average tumor volume was less than 20, 000 mm^3^, with no diameter exceeding 20 mm. We obtained twenty-four female BALB/c mice (6 weeks old) from Shanghai Model Organisms Center, Inc. (China). Mice were randomly assigned to two groups and injected subcutaneously with 1×10^6^ Renca cells harboring either sh-NC or sh-LINC00887 to form syngeneic tumor models. One week later, 12 mice from each group were reassigned into two subgroups and treated with lentiviruses encoding sh-NC or sh-UPF1. Tumor volume was documented every 7 days. Upon completion of the 5-week observation period, mice were humanely euthanized; tumors were extracted, weighed, and stored for further analysis. At the end of the experiment, mice were anesthetized by inhalation of 3% isoflurane and then euthanized by cervical dislocation.

### 2.17 Immunohistochemistry

Fixed in 4% paraformaldehyde (P1110, Solarbio, China), mouse tumor tissues were prepared into 4-micron paraffin sections. Tissue sections underwent deparaffinization and rehydration before heat-induced epitope retrieval with commercial citrate buffer (P0083, Beyotime, China). Endogenous peroxidase was blocked for 10 min utilizing peroxidase blocking solution (P0100A, Beyotime, China) at room temperature. Non-specific binding sites were blocked utilizing goat serum before incubation at 4 °C overnight with rabbit primary antibodies against LDH-A (ab52488), UPF1 (ab109363), and Ki67 (ab15580, abcam, UK). After PBS rinsing, sections were exposed to goat anti-rabbit IgG (ab6721, abcam, UK) for 30 min at 37 °C. Following DAB development (P0203, Beyotime, China) and hematoxylin counterstaining (C0107, Beyotime, China), slides were dehydrated, cleared, and mounted, and then visualized under an Olympus microscope from Japan.

### 2.18 Immunofluorescence

Fixed mouse tumor tissues were sectioned, dewaxed, rehydrated, and subjected to antigen retrieval. Following peroxidase inhibition and blocking procedures, tissue sections were exposed to rabbit anti-CD206 (ab64693, Abcam, UK) overnight at 4 °C. Unbound antibodies were washed off the next day. Subsequently, an one-h incubation at room temperature was performed using goat anti-rabbit IgG secondary antibody conjugated with Alexa Fluor 488 (ab150077, Abcam, UK) in the dark. DAPI-containing anti-fade mounting medium (P0131, Beyotime, China) was applied to the tissue sections, which were then coverslipped. Fluorescent signals were visualized using a fluorescence microscope (Olympus, Japan).

### 2.19 Data processing

Statistical analysis and data visualization were conducted on GraphPad Prism 8.0. Each experiment was performed in triplicate. Pearson’s correlation analysis was conducted to evaluate linear correlations, with statistical significance defined as *p* < 0.05 and Pearson’s R>0.30. Results are shown as mean ± standard deviation. Two-group comparisons were performed using Student’s t-test, and one-way analysis of variance followed by Tukey’s *post-hoc* test was used for comparisons among multiple groups. *p* < 0.05 indicated statistically significant.

## 3 Results

### 3.1 LINC00887 is highly expressed and its impact on aerobic glycolysis in ccRCC

In this study, we discuss the relationship between LINC00887 and UPF1, and whether it is involved in reprogramming the metabolic pathways of ccRCC cells, leading to lactate buildup, which in turn promotes macrophages to an M2 phenotype and immune evasion of the tumor. The overall study design is depicted in the workflow chart (**Figure 1**). To examine the expression profile of LINC00887 in ccRCC, we employed the TCGA-KIRC dataset and compared the transcriptional levels of LINC00887 between tumor tissues and adjacent normal samples. A significant upregulation of LINC00887 mRNA was observed in ccRCC tissues compared to normal controls, and its expression level was markedly elevated alongside the increasing malignancy degree of tumors (**Figure 2A**; **Supplementary Figures 1A–D**). qRT-PCR validation further confirmed elevated LINC00887 mRNA in ccRCC cells compared to normal renal cells (**Figure 2B**). To elucidate the functional role of LINC00887 in ccRCC progression, we conducted KEGG pathway analysis, which identified its significant enrichment in key oncogenic pathways, including glycolysis, HIF-1 signaling, and PPAR signaling pathways. Notably, glycolysis emerged as the most significantly enriched pathway (**Figures 2C, D**). Bioinformatic methods unraveled a significant positive correlation between LINC00887 levels and glycolytic gene signatures in ccRCC (**Figure 2E**). ccRCC has been identified as a malignant tumor characterized significantly by changes in glucose metabolism (19).

**Figure 1:**
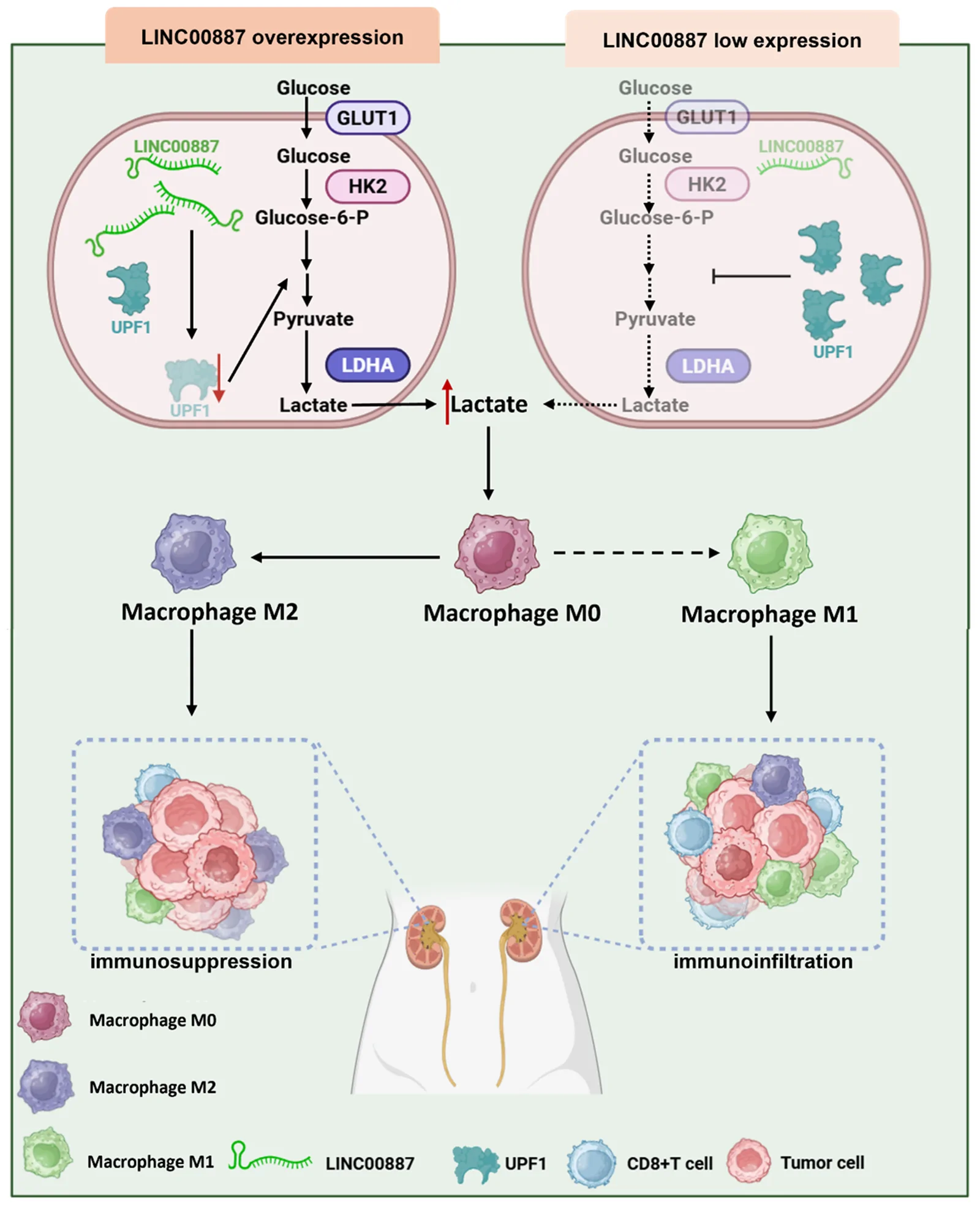
Workflow chart.

**Figure 2:**
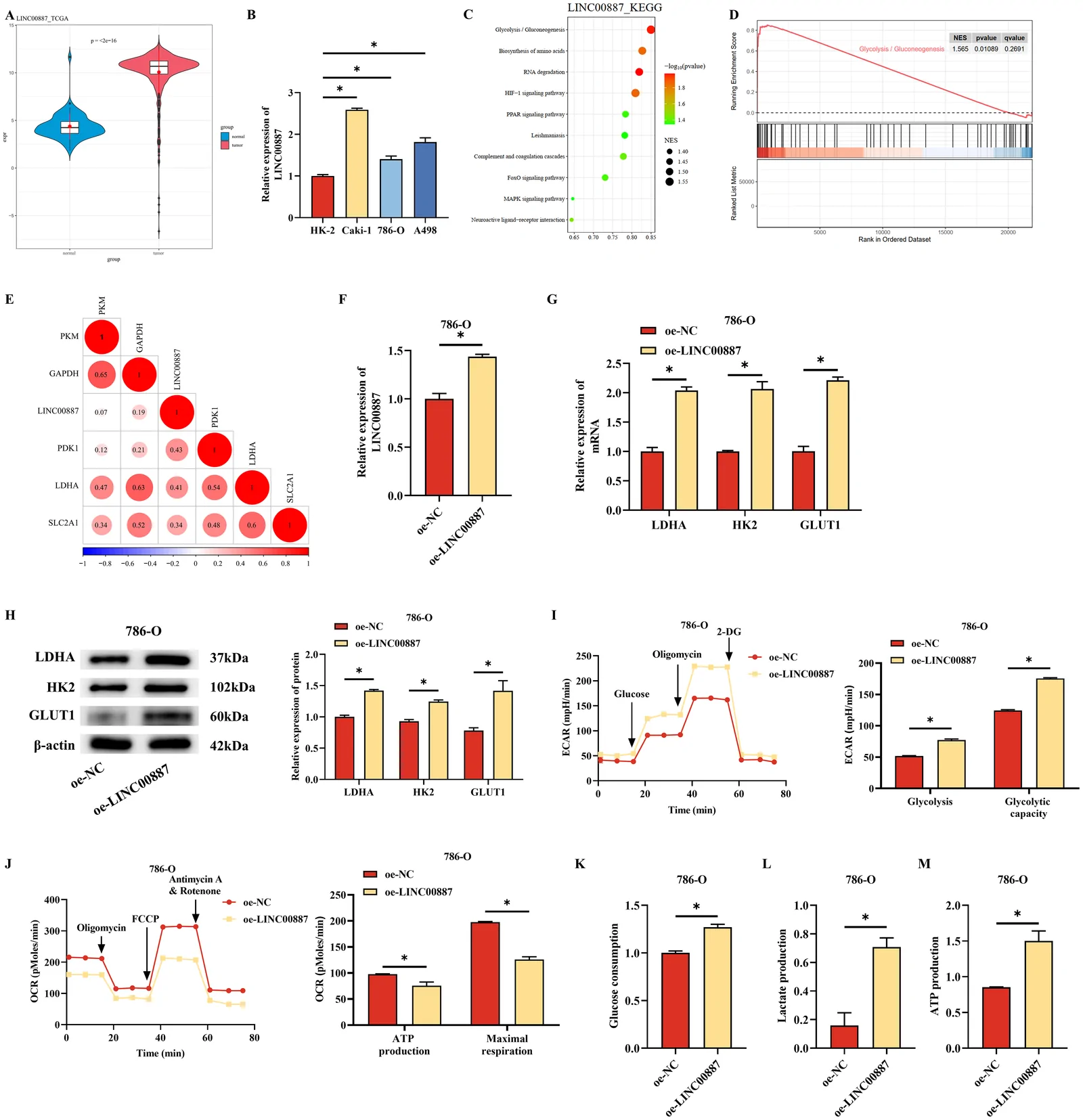
LINC00887 is highly expressed and its impact on aerobic glycolysis in ccRCC, **(A)** Analysis of LINC00887 mRNA levels in ccRCC and adjacent normal tissues from TCGA-KIRC dataset. **(B)** qRT-PCR test of LINC00887 mRNA levels in HK-2, 786-O, Caki-1, and A498 cell lines. **(C)** KEGG pathway analysis of signaling pathways enriched by LINC00887 in ccRCC. **(D)** KEGG analysis showing enrichment of LINC00887 in the glycolysis pathway in ccRCC. **(E)** Pearson correlation analysis between LINC00887 and glycolytic marker genes. 786-O cells were transfected with either oe-NC or oe-LINC00887, respectively. **(F)** qRT-PCR was performed to verify transfection efficiency. **(G)** qRT-PCR measurement of glycolytic marker genes LDH-A, HK2, and GLUT1 mRNA levels in each group. **(H)** immunoblotting analysis of glycolytic marker LDH-A, HK2, and GLUT1 protein levels in each group. **(I)** Seahorse metabolic analyzer was used to measure ECAR in each group, calculating glycolytic value and glycolytic capacity. **(J)** Seahorse metabolic analyzer was used to measure OCR in each group, calculating ATP production, and maximal respiration. **(K–M)** Kits were used to measure glucose consumption **(K)**, lactate production **(L)**, and ATP yield **(M)**. *indicates *p* < 0.05. ccRCC, Clear cell renal cell carcinoma; qRT-PCR, Quantitative reverse transcription polymerase chain reaction; KEGG, Kyoto Encyclopedia of Genes and Genomes; LDH-A, Lactate dehydrogenase A; HK2, Hexokinase 2; GLUT1, Glucose transporter 1; ECAR, Extracellular acidification rate; OCR, Oxygen consumption rate; ATP, Adenosine triphosphate.

For subsequent functional studies, we selected two representative ccRCC cell lines, Caki-1 and 786-O, which are collectively referred to as ccRCC cells throughout this research. Modulation of LINC00887 expression (via overexpression or siRNA knockdown) in ccRCC cells enabled assessment of its role in glycolytic metabolism (**Figure 2F**; **Supplementary Figure 2A**). LDH-A, HK2 and GLUT1 have been identified as markers of aerobic glycolysis (20). Ectopic overexpression of LINC00887 significantly upregulated both mRNA and protein levels of LDH-A, HK2 and GLUT1 in ccRCC cells, whereas LINC00887 knockdown produced the opposite effect (**Figures 2G, H**; **Supplementary Figures 2B, C**). In cellular energy metabolism, the Oxygen Consumption Rate (OCR) serves as a direct indicator of oxidative phosphorylation (OXPHOS) activity, while the Extracellular Acidification Rate (ECAR) quantifies glycolytic flux by measuring the pH decrease resulting from lactate accumulation in the extracellular medium (21). Overexpression of LINC00887 significantly increased ECAR while suppressing OCR in ccRCC cells, indicating a metabolic shift toward glycolysis. Conversely, LINC00887 knockdown attenuated glycolytic flux, as demonstrated by reduced ECAR and elevated OCR (**Figures 2I, J**; **Supplementary Figures 2D, E**). Consistent with the ECAR/OCR findings, LINC00887-overexpressing cells exhibited significantly increased glucose uptake, lactate secretion, and ATP production. Conversely, LINC00887 knockdown markedly attenuated these metabolic parameters, reducing glucose consumption, lactate output, and ATP levels (**Figures 2K–M**; **Supplementary Figures 2F–H**). These results demonstrate that LINC00887 drives metabolic reprogramming in ccRCC by promoting aerobic glycolysis. LINC00887 promotes lactate accumulation via aerobic glycolysis, thereby driving macrophage M2 polarization in ccRCC.

Via metabolic reprogramming, most notably elevated glycolytic activity, tumor cells produce abundant lactate that accumulates within tissues, thereby establishing an acidic tumor microenvironment. This acidosis impairs cytotoxic T cell function while encouraging polarization of tumor-associated macrophages toward an immunosuppressive M2 phenotype, ultimately fostering tumor immune evasion (22, 23). To further characterize the immunoregulatory function of LINC00887, we comprehensively analyzed its association with immune cell infiltration patterns and macrophage polarization status in the tumor microenvironment. The results revealed a significant positive correlation between LINC00887 and M2 macrophages (**Supplementary Figures 3A, B**). To further examine whether LINC00887 could drive M2 macrophage polarization by accumulating lactate through aerobic glycolysis, we treated 786-O cells overexpressing oe-NC or oe-LINC00887 with PBS or Oxamate (OXM), an LDH-A inhibitor. The supernatant was collected and applied to culture M0 macrophages for differentiation assessment. We cultured macrophages with supernatants from cells overexpressing LINC00887 and obseved an evident elevation of M2 macrophage markers Arg1, Fizz1, and IL-10, and a downregulation of M1 markers iNOS, TNF-α, and IL-1β. Notably, this LINC00887-mediated macrophage M2 polarization was effectively abrogated by OXM treatment (**Supplementary Figures 3C, D**). Flow cytometry revealed that the overexpression of LINC00887 in 786-O cells drives macrophage polarization towards an M2 phenotype, as evidenced by the increased proportion of CD68^+^CD206^+^ cells and decreased CD68^+^CD86^+^ cells, this polarization trend was mitigated by OXM, which limits lactate generation, a hallmark of glycolytic activity (**Supplementary Figures 3E, F**). These data suggest that LINC00887 may direct macrophage polarization in ccRCC by enhancing glycolysis and lactate accumulation.

### 3.2 LINC00887 physically interacts with UPF1, and their expression levels show a significant negative correlation

To clarify the precise mechanisms by which LINC00887 regulates aerobic glycolysis in ccRCC, we conducted a bioinformatics analysis to forecast its downstream target proteins. The analysis identified UPF1 as a potential target of LINC00887, with a pronounced negative correlation between them as determined by Pearson correlation analysis (**Figure 3A**). Subsequently, we examined UPF1 expression by qRT-PCR and immunoblotting, and found that LINC00887 knockdown upregulated both mRNA and protein levels of UPF1 in ccRCC cells (**Figures 3B, C**). The binding of LncRNA to proteins is one of the important mechanisms by which it exerts its biological functions (24). RIP analysis has uncovered an interaction between UPF1 and LINC00887. When UPF1 levels were reduced, there was a corresponding decrease in LINC00887 binding, and increasing UPF1 had the opposite effect (**Figure 3D**). To investigate the mechanism by which LINC00887 negatively regulates UPF1, Caki-1 cells transfected with sh-NC or sh-LINC00887 were treated with the proteasome inhibitor MG132 (10 μM, HY-13259, MCE, USA) for 6 h, followed by Western blot analysis of UPF1 protein expression. The results showed that the increase in UPF1 protein levels in the sh-LINC00887 group was not significantly affected by MG132 (**Figure 3E**). These findings indicate that the negative regulation of UPF1 by LINC00887 is not mediated through the proteasomal degradation pathway but rather occurs primarily at the transcriptional level.

**Figure 3:**
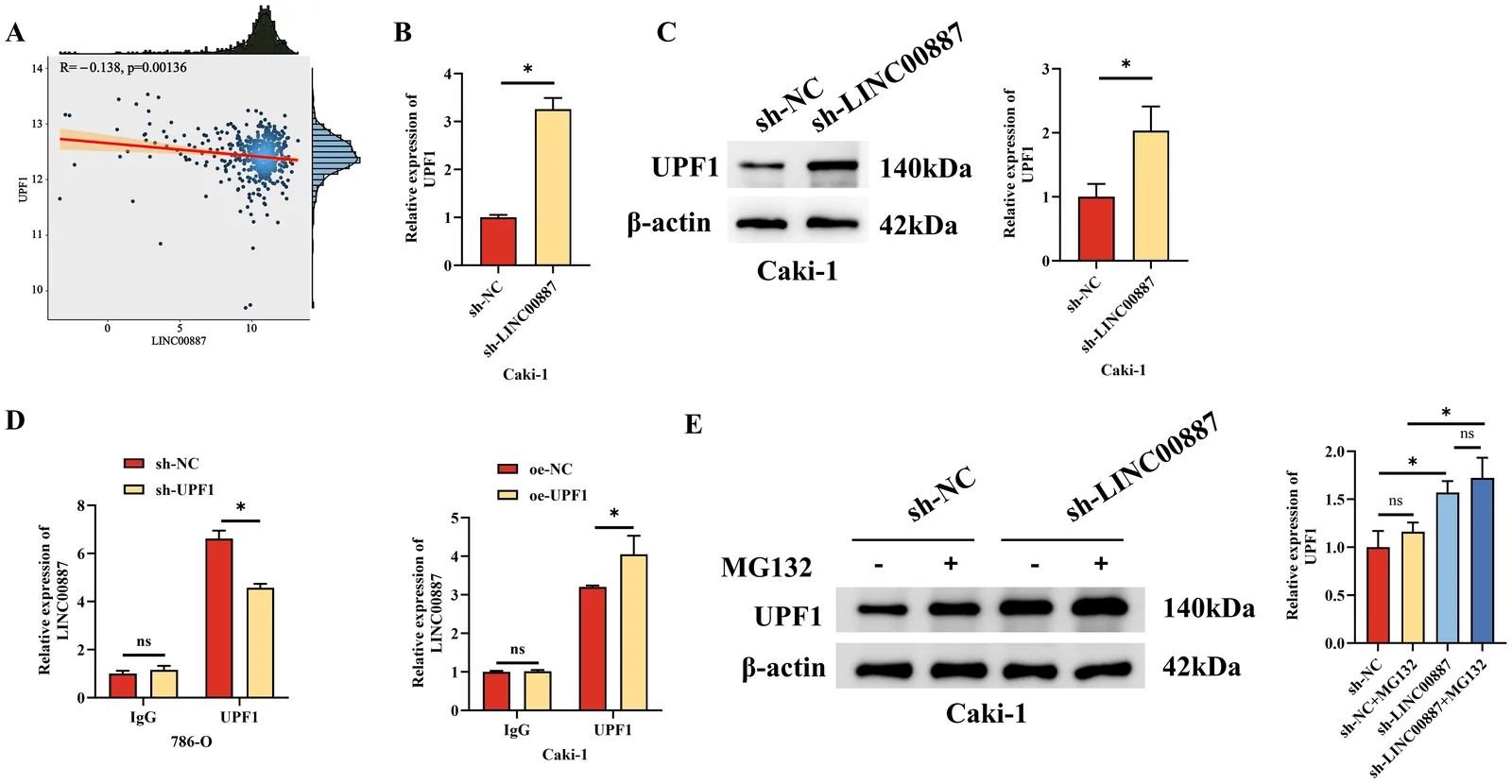
LINC00887 interacts with UPF1, **(A)** Pearson correlation analysis between LINC00887 and UPF1. Caki-1 cells were transfected with sh-NC or sh-LINC00887, **(B)** UPF1 mRNA levels assayed by qRT-PCR. **(C)** UPF1 protein expression was tested by immunoblotting. **(D)** 786-O cells were transfected with sh-NC or sh-UPF1, and Caki-1 cells with oe-NC or oe-UPF1, RIP assays performed to verify the binding of LINC00887 to UPF1. ns, no significant difference; **(E)** Caki-1 cells stably expressing sh-NC or sh-LINC00887 were treated with the proteasome inhibitor MG132 (10 μM) for 6 h, and UPF1 protein levels were examined by Western blot. *,*p* < 0.05. UPF1, Up-frameshift protein 1; qRT-PCR, Quantitative reverse transcription polymerase chain reaction.

### 3.3 Distribution and metabolic impact of UPF1 in ccRCC

We utilized scRNA-seq to delineate the expression profile of UPF1 across ccRCC samples. The dataset comprised two tumor samples and two adjacent tissues, amassing a total of 40, 474 cells (**Figures 4A, B**). After applying quality control filters, we selected 39, 518 cells for further analysis. We then employed clustering algorithms to categorize cells into 16 groups based on their gene expression profiles, which were visualized via UMAP and tSNE (**Figures 4C, D**). Following the clustering analysis, we annotated the cells according to their unique gene expression patterns, classifying seven distinct cell subtypes: “Fibroblasts, “ “B cells, “ “Endothelial cells, “ “Epithelial cells, “ “T/NK cells, “ “Plasmablasts, “ and “Monocytes/Macrophages” (**Figure 4E**). The bubble plot depicted the clustering results and the expression of marker genes (**Figure 4F**). We further analyzed the proportions of cellular subpopulations in both tumor tissues and adjacent normal tissues. We identified a decrease in fibroblasts and an increase in T/NK cells and macrophages (**Figure 4G**).

**Figure 4:**
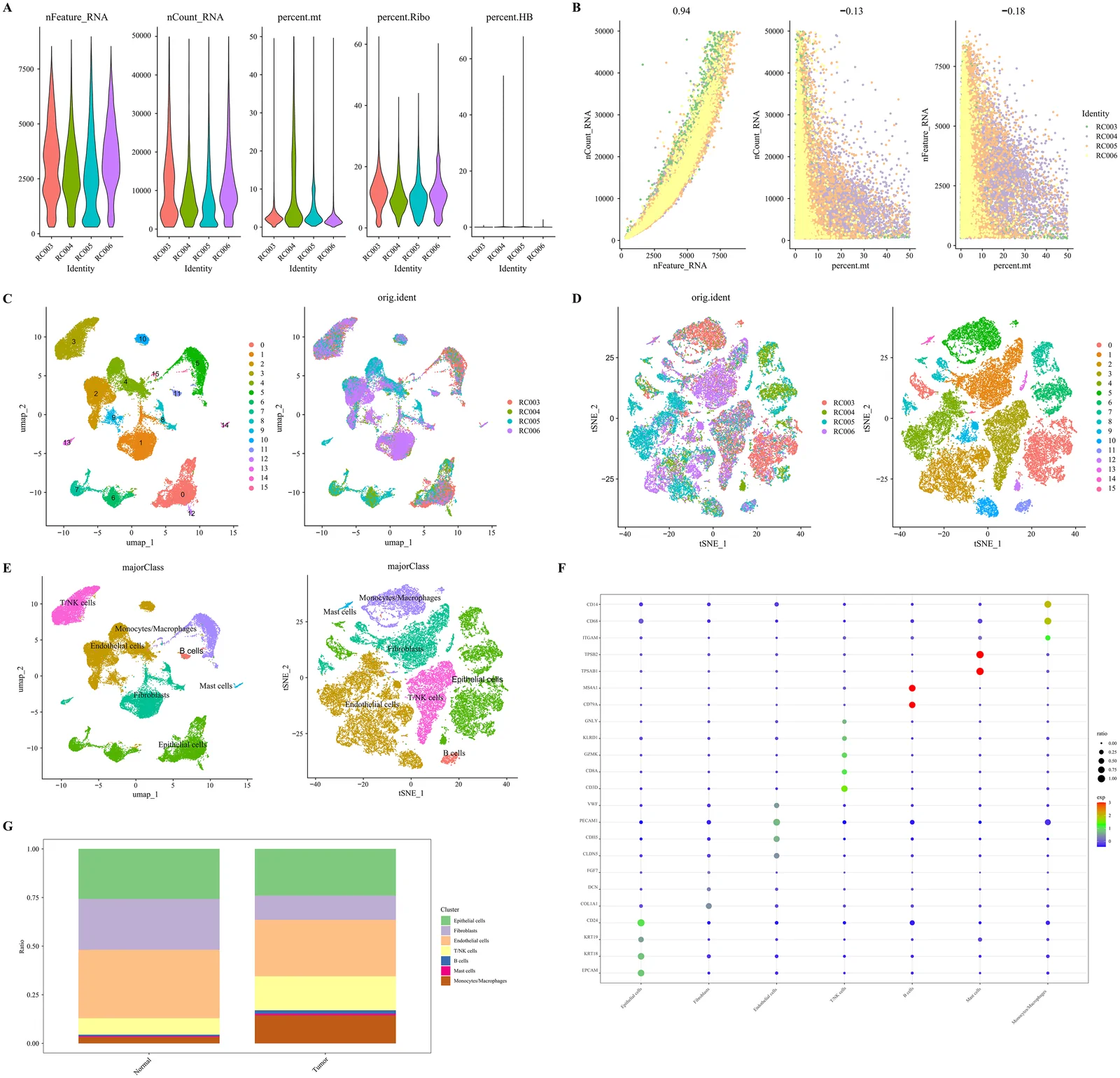
Overview of scRNA-seq derived from ccRCC and adjacent tumor tissues, **(A, B)** Quality control plots for ribosomal genes, filtered features, mitochondrial genes, counts, and red blood cell genes, with selection criteria of nFeature_RNA < 10, 000 & nCount_RNA < 50, 000 & percent.mt < 50. Umap **(C)** and tSNE **(D)** plots of cell clustering results. **(E)** Umap (left) and tSNE (right) plots for the 7 main cell subtypes. **(F)** Bubble plot of markers for the 7 main cell subtypes. **(G)** Proportions of seven major cell subtypes in ccRCC tumor tissues versus adjacent normal tissues. ccRCC, Clear cell renal cell carcinoma.

Leveraging scMetabolism to scrutinize the metabolic pathways listed in KEGG, we uncovered metabolic pathway irregularities within the tumor tissue, especially regarding glycolysis and fatty acid metabolism (**Supplementary Figure 4A**). Delving into metabolic disparities among the seven cell types, we discovered that these metabolic anomalies mainly occurred in epithelial cells (**Supplementary Figure 4B**). We also found considerable variation in UPF1 expression, especially within the epithelial cells of the tumor compared with the surrounding tissue (**Supplementary Figure 4C**), prompting us to concentrate our subsequent analysis on epithelial cells (**Supplementary Figure 4D**), where we employed the epithelial markers EPCAM and KRT18 for confirmation (**Supplementary Figures 4E, F**). The expression pattern of UPF1 was highly different in ccRCC tumor tissues in comparison to adjacent tissues (**Supplementary Figure 4G**). In addition, KEGG pathway enrichment analysis revealed that UPF1 exhibited prominent enrichment in glycolysis-related pathways (**Supplementary Figure 4H**). The results of intercellular communication analysis revealed that both the number and strength of cell-cell interactions were markedly elevated in ccRCC tumor tissues relative to adjacent normal tissues (**Supplementary Figure 4I**), indicating robust signal crosstalk within the tumor microenvironment. Overall, the scRNA-seq analysis suggests that UPF1 expression differed between ccRCC tumors and normal epithelial cells, highlighting its relevance to glycolytic metabolism.

### 3.4 UPF1 exhibits low expression in ccRCC and functions as a tumor suppressor by inhibiting malignant progression

Bioinformatics analysis of the TCGA-KIRC dataset revealed a remarkable downregulation in UPF1 mRNA in tumor tissues (**Figure 5A**). Furthermore, the expression level of UPF1 decreased significantly with the escalation of tumor malignancy, and high UPF1 expression was correlated with favorable survival outcomes in patients (**Supplementary Figures 5A–F**). This was supported by qRT-PCR and immunoblotting results in ccRCC cell lines, which showed a notable reduction in UPF1 mRNA and protein levels (**Figures 5B, C**). Subsequently, we manipulated UPF1 levels through targeted knockdown or overexpression in ccRCC cells. Through CCK-8 and colony formation, we observed that silencing UPF1 notably boosted 786-O cell viability and proliferation; in contrast, overexpression of UPF1 markedly reduced the vitality and proliferative potential of Caki-1 cells (**Figures 5D, E**). Moreover, our cell scratch and Transwell assays revealed that silencing UPF1 in ccRCC cells significantly promoted their migration and invasion capabilities, but its overexpression exerted the opposite effect, curbing these properties (**Figures 5F, G**). Flow cytometry further showed that reducing UPF1 levels led to reduced apoptotic rates and induced cell cycle arrest, whereas UPF1 overexpression promoted apoptosis and disturbed cell cycle (**Figures 5H, I**). Thus, UPF1 could act as a tumor repressor, influencing the aggressive behavior of ccRCC.

**Figure 5:**
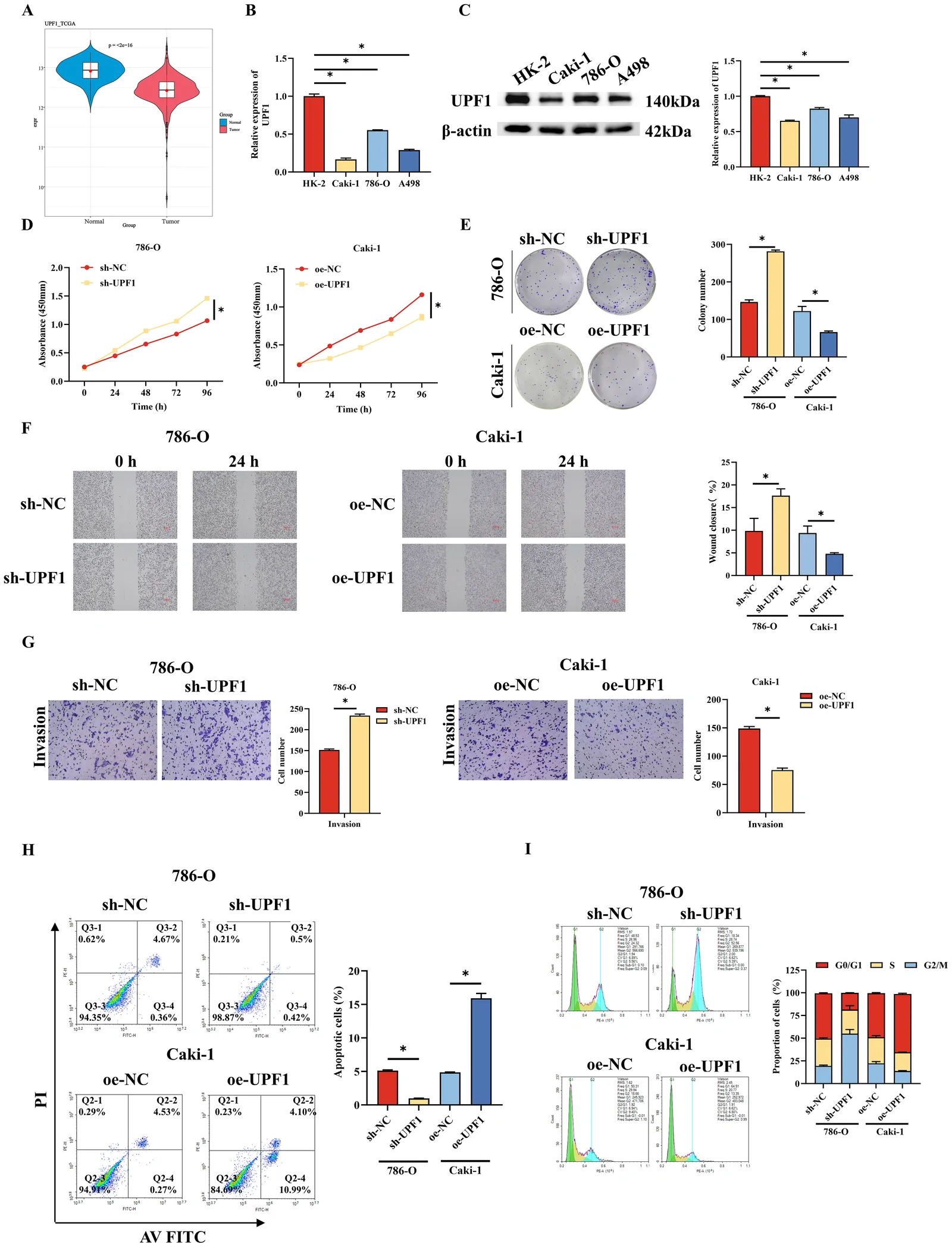
LINC00887-mediated UPF1 suppresses ccRCC malignant progression, **(A)** mRNA levels of UPF1 in ccRCC tumor tissues versus adjacent normal tissues as analyzed by TCGA-KIRC dataset. **(B)** qRT-PCR detection of UPF1 mRNA in HK-2, Caki-1, 786-O, and A498 cells. **(C)** Immunoblotting assessment of UPF1 protein in renal cell lines. **(D)** CCK-8 assay for cell viability in 786-O cells expressing sh-NC or sh-UPF1 and Caki-1 cells expressing oe-NC or oe-UPF1. **(E)** Colony formation assay determined cell proliferation in the transfected cells. **(F)** Scratch assay evaluated cell migration. **(G)** Transwell assessed cell invasion. **(H)** Flow cytometry analyzed apoptosis in different cell groups. **(I)** Flow cytometry examined cell cycle distribution in various cell groups. *, *p* < 0.05. ccRCC, Clear cell renal cell carcinoma; UPF1, Up-frameshift protein 1. qRT-PCR, Quantitative reverse transcription polymerase chain reaction; TCGA, The cancer genome atlas; KIRC, Kidney renal clear cell carcinoma.

### 3.5 LINC00887 modulates aerobic glycolysis through UPF1 in ccRCC

To determine whether LINC00887 affects aerobic glycolysis of ccRCC by targeting UPF1, we performed the following experiments. qRT-PCR and immunoblotting showed that LINC00887 knockdown increased UPF1 expression in tumor cells and suppressed the expression of LDH-A, HK2, and GLUT1. However, UPF1 knockdown upregulated the expression levels of core proteins in aerobic glycolytic pathways such as LDH-A, HK2 and GLUT1. (**Figures 6A, B**; **Supplementary Figures 6A, B**). UPF1 knockdown counteracted the impacts of LINC00887 knockdown on glycolytic capacity, ATP production, and maximal respiration in ccRCC cells (**Figures 6C, D**; **Supplementary Figures 6C, D**), with corresponding changes in glucose consumption, lactate, and ATP (**Figures 6E–G**; **Supplementary Figures 6E–G**). Collectively, LINC00887 may target UPF1 to modulate aerobic glycolysis in ccRCC.

**Figure 6:**
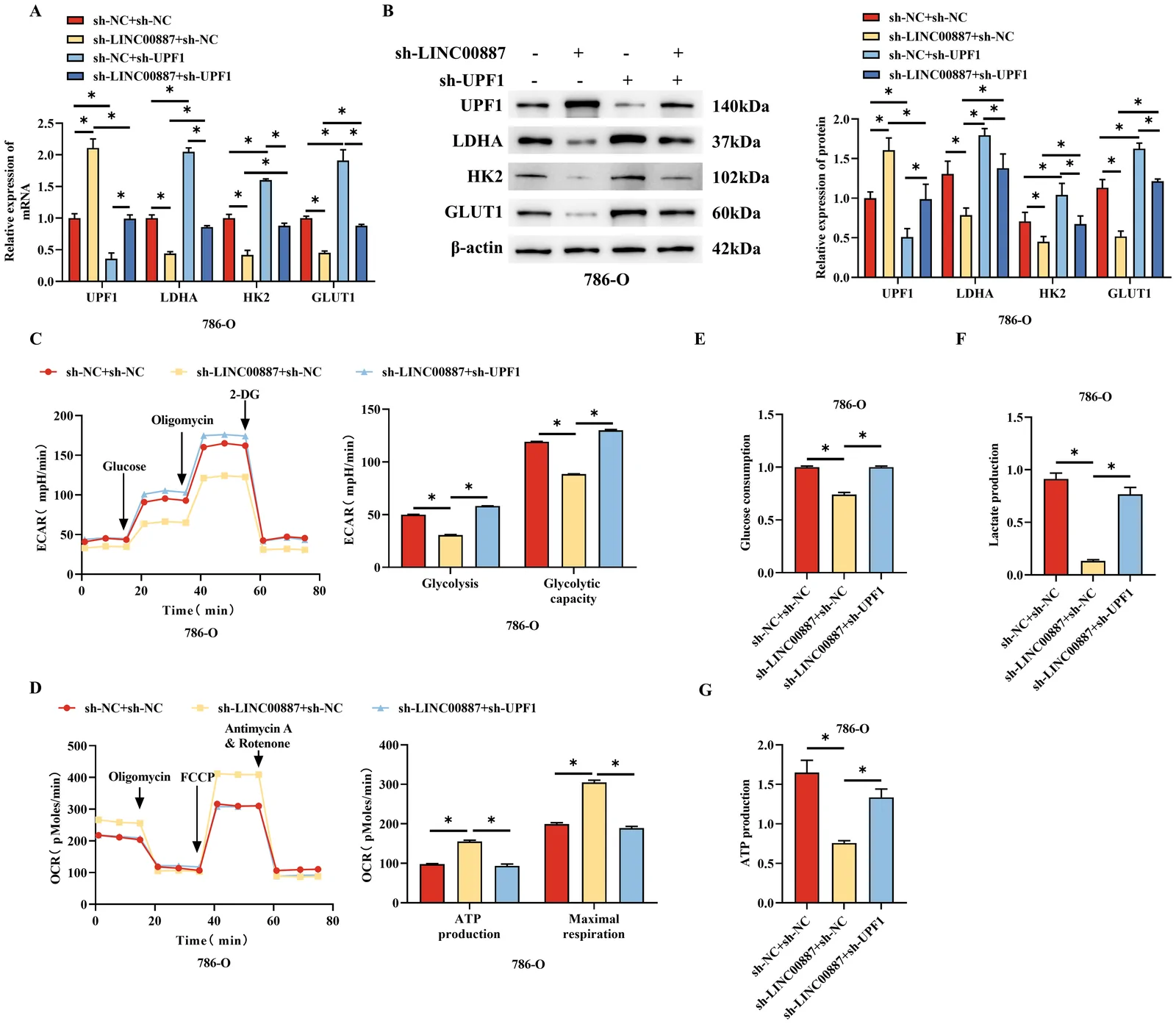
LINC00887 regulates aerobic glycolysis by interacting with UPF1 in ccRCC, 786-O cells were transfected with sh-NC+sh-NC, sh-LINC00887+sh-NC, sh-NC+sh-NC+sh-UPF1, or sh-LINC00887+sh-UPF1. **(A)** qRT-PCR tested UPF1, LDH-A, HK2, and GLUT1 mRNA levels in each group of cells. **(B)** Immunoblotting assessed the protein levels of UPF1, LDH-A, HK2, and GLUT1 within each group of cells. sh-NC + sh-NC, sh-LINC00887 + sh-NC, or sh-LINC00887 + sh-UPF1 was transfected into 786-O cells, respectively, **(C)** A Seahorse Energy Metabolism Analyzer was utilized to measure the ECAR of each group of cells, calculating glycolytic values, glycolytic capacity, and glycolytic reserve. **(D)** OCR of cells from each group assessed by the Seahorse Energy Metabolism Analyzer, based on basal respiration, ATP production, and maximal respiration. Kit assays were conducted to measure glucose consumption **(E)**, lactate production **(F)**, and ATP production **(G)**. *p < 0.05. ccRCC, Clear cell renal cell carcinoma; UPF1, Up-frameshift protein 1; qRT-PCR, Quantitative reverse transcription polymerase chain reaction; LDH-A, Lactate dehydrogenase A; HK2, Hexokinase 2; GLUT1, Glucose transporter 1; ECAR, Extracellular acidification rate; OCR, Oxygen consumption rate; ATP, Adenosine triphosphate.

### 3.6 UPF1 inhibits macrophage M2 polarization by regulating ccRCC aerobic glycolysis

Tumor cells produce lactic acid through glycolysis, forming lactic acid-rich TME, which affects macrophages. Lactic acid induces M2 polarization-related gene expression in macrophages, promotes polarization of macrophages towards the M2 immunosuppressive type, and thereby promotes cancer growth. We observed that UPF1 knockdown in ccRCC cells not only decreased its protein levels but also led to an increase in glycolytic markers like LDH-A, HK2, and GLUT1 by qRT-PCR and immunoblotting. The application of the glycolytic inhibitor 2-DG normalized these protein levels (**Figure 7A**). Moreover, 2-DG treatment also reversed the enhanced glycolytic phenotype and the reduction in maximal oxygen consumption caused by UPF1 knockdown in ccRCC cells (**Figures 7B, C**), aligning with changes in glucose uptake, lactate secretion, and ATP production (**Figures 7D–F**). Overexpression of UPF1 suppressed glycolysis-associated proteins and glycolytic flux levels, and these changes were partially reversed after GLUT1 overexpression (**Supplementary Figures 7A–F**).

**Figure 7:**
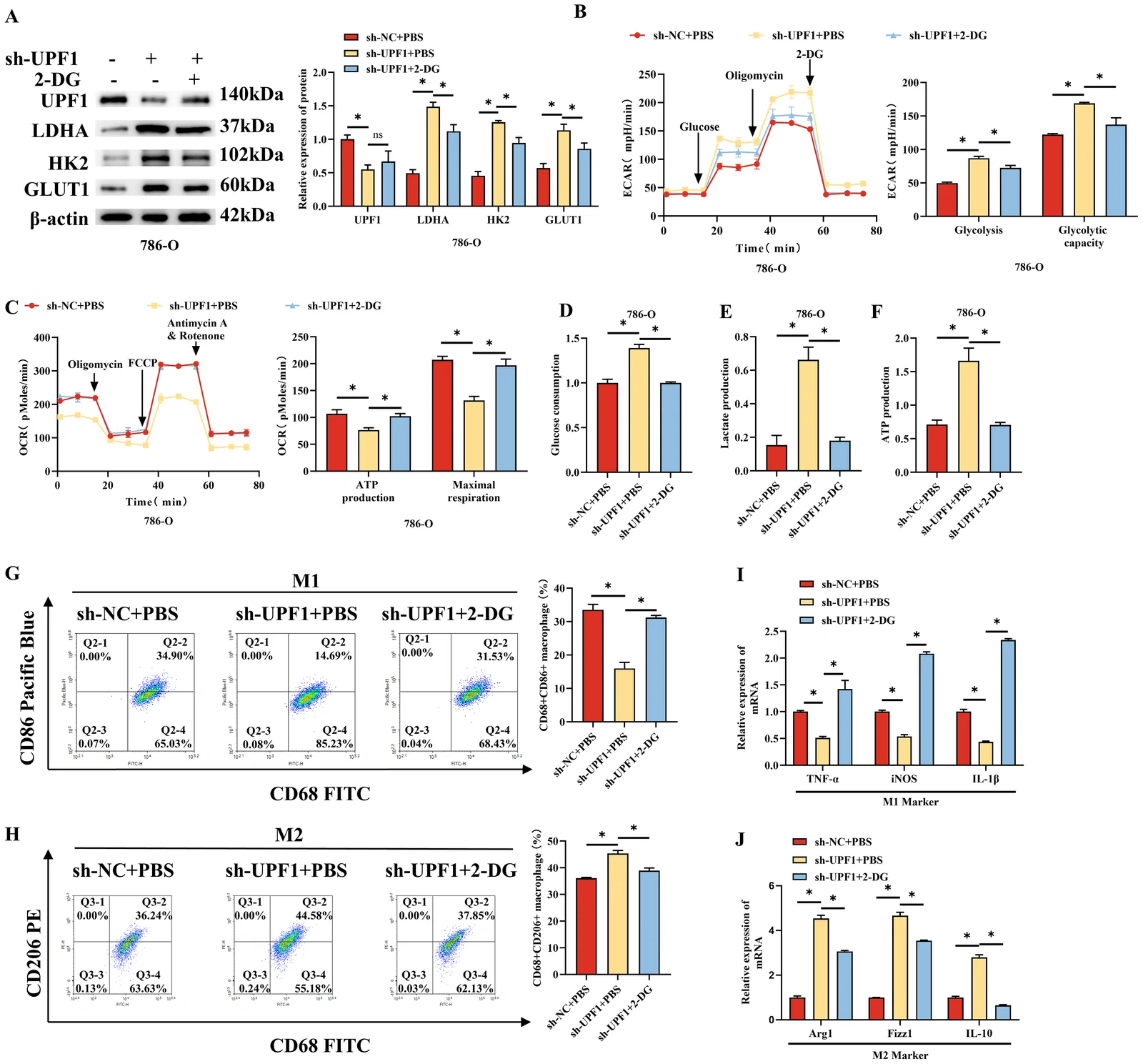
UPF1 regulates aerobic glycolysis in ccRCC to inhibit macrophage M2 polarization, 786-O cell groups of sh-NC+PBS, sh-UPF1+PBS, or sh-UPF1 + 2-DG. **(A)** Immunoblotting detected protein levels of UPF1, LDH-A, HK2, and GLUT1 in each group of cells. **(B)** Seahorse Extracellular Flux Analyzer tested ECAR of each group, calculating glycolytic rate, glycolytic capacity, and glycolytic reserve. **(C)** Seahorse Extracellular Flux Analyzer tested OCR, calculating basal respiration, ATP production, and maximum respiration. **(D–F)** Assay kits for glucose consumption **(D)**, lactate production **(E)**, and ATP production **(F)**. Conditioned medium from differently treated Caki-1 cells was used to culture M0 macrophages, **(G, H)** Flow cytometry quantification of M1 macrophages (CD68^+^CD86^+^) and M2 macrophages (CD68^+^CD206^+^). **(I, J)** qRT-PCR analysis of M1 markers [TNF-α, iNOS, IL-1β; **(I)**] and M2 markers [Arg1, Fizz1, IL-10; **(J)**]. *, *p* < 0.05. cRCC, Clear cell renal cell carcinoma; UPF1, Up-frameshift protein 1; 2-DG, 2-Deoxy-D-glucose; LDH-A, Lactate dehydrogenase A; HK2, Hexokinase 2; GLUT1, Glucose transporter 1; ECAR, Extracellular acidification rate; OCR, Oxygen consumption rate; ATP, Adenosine triphosphate; CD68, Cluster of differentiation 68; CD86, Cluster of differentiation 86; CD206, Mannose receptor C-type 1; TNF-α, Tumor necrosis factor-alpha; iNOS, Inducible nitric oxide synthase; IL-1β, Interleukin - 1β; Arg1, Arginase 1; Fizz1, Found in inflammatory zone 1; IL-10, Interleukin -10.

We assessed whether UPF1-mediated regulation of aerobic glycolysis influences macrophage polarization by exposing M0 macrophages to conditioned media from ccRCC cells with modulated UPF1 expression. Flow cytometry analysis revealed that downregulation of UPF1 in ccRCC cells promoted M0-to-M2 macrophage polarization. This effect was abrogated by 2-DG treatment, which additionally induced a significant shift toward the M1 phenotype (**Figures 7G, H**). Analyzing M1 and M2 macrophage marker levels by qRT-PCR, we discovered that 2-DG treatment counteracted the reduction in M1 macrophage marker expression induced by UPF1 knockdown and impressively decreased the expression of M2 macrophage markers (**Figures 7I, J**). Similarly, UPF1 overexpression promoted M1 macrophage polarization while suppressing M2 polarization, and this trend was reversed by further upregulation of GLUT1 (**Supplementary Figures 7G–J**). These data suggest that UPF1 might regulate aerobic glycolysis in ccRCC and inhibit M2 polarization of macrophages.

### 3.7 Overexpression of UPF1 in ccRCC inhibits M2 macrophage polarization and enhances the tumor suppressor function of CD8^+^ T cells

There exists a complex immune regulatory relationship between macrophage polarization and CD8^+^ T cell activation (25). To investigate how UPF1 regulates the aerobic glycolysis pathway, where lactate induces macrophage polarization, thereby affecting CD8^+^ T cell activity, we manipulated UPF1 levels in 786-O cells and cultured M0 macrophages with the resulting conditioned media. When these macrophages were cultured with activated CD8^+^T cells, we observed that the sh-UPF1+PBS treatment group led to reduced CD8^+^ T cell proliferation and increased apoptosis. Conversely, the sh-UPF1 + 2-DG treatment group showed a reversal of these effects (**Figures 8A, B**). In ccRCC cells, silencing UPF1 disrupted M2 macrophage polarization, which typically suppresses CD8^+^ T cell activity. However, when glycolysis was inhibited using 2-DG, CD8^+^ T cell cytotoxicity was enhanced (**Figure 8C**). This suggests that UPF1 could be a central regulator in ccRCC, promoting the antitumor activity of CD8^+^ T cells by preventing M2 macrophage polarization.

**Figure 8:**
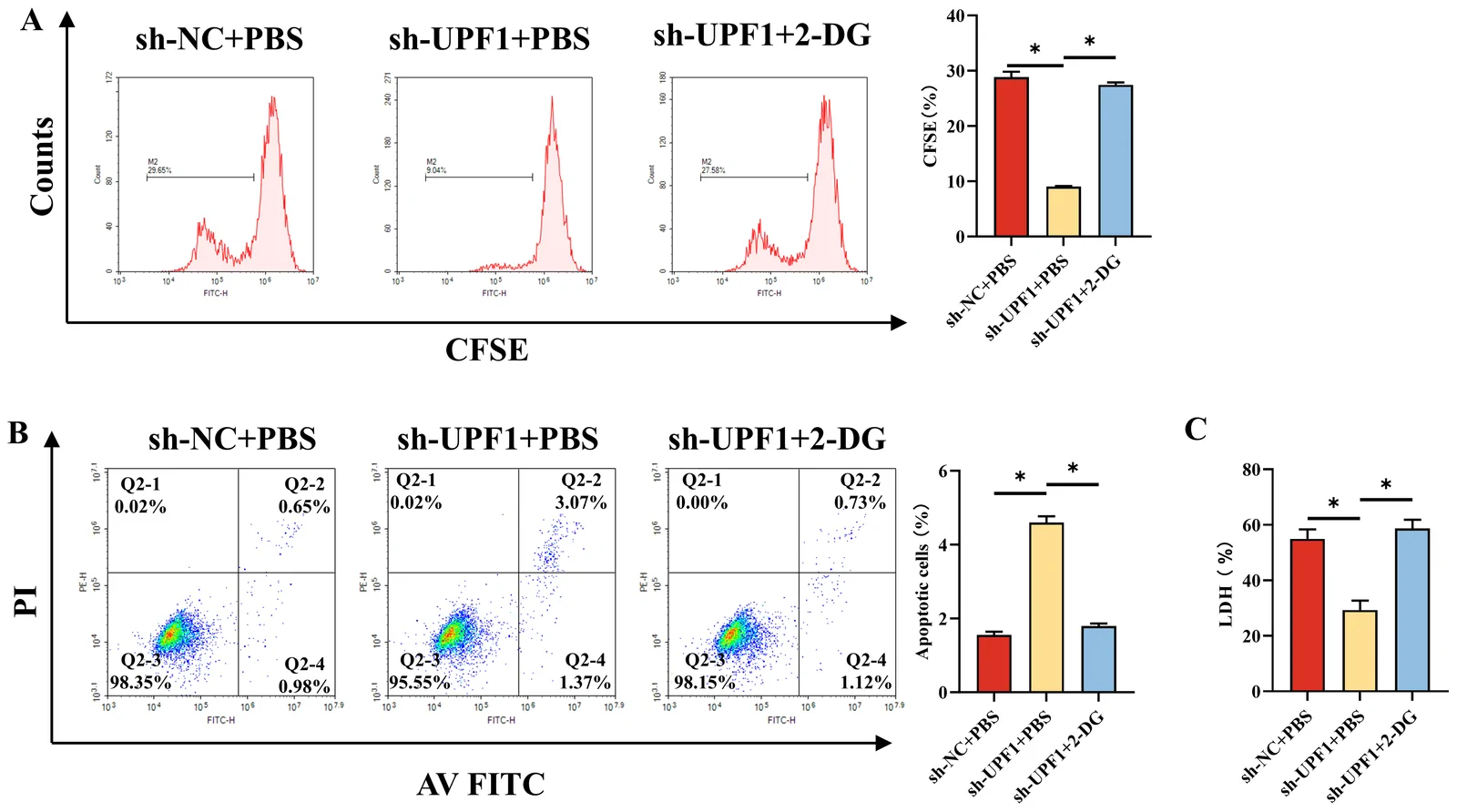
Overexpression of UPF1 in ccRCC inhibits M2 macrophage polarization and enhances the tumor suppressor function of CD8^+^ T cells, Caki-1 cell groups of sh-NC+PBS, sh-UPF1+PBS, or sh-UPF1 + 2-DG, and the conditioned medium was used to culture and polarize M0 macrophages. The polarized macrophages were cultured with activated CD8^+^ T cells. **(A)** CFSE assessed CD8^+^ T cell proliferative capacity. **(B)** Flow cytometry measured apoptosis rate of CD8^+^ T cells in different co-culture systems. **(C)** CD8^+^ T cells from culture systems were co-cultured with normal Caki-1 cells, with LDH assays determining cytotoxic effect of CD8^+^ T cells. *, *p* < 0.05. cRCC, Clear cell renal cell carcinoma; UPF1, Up-frameshift protein 1; 2-DG, 2-Deoxy-D-glucose; CFSE, Carboxyfluorescein succinimidyl ester.

### 3.8 LINC00887/UPF1 mediated aerobic glycolysis leads to lactic acid accumulation and facilitates immune evasion in ccRCC *in vivo*

To ensure *in vitro* results held *in vivo*, we developed mouse RCC xenograft models using cells expressing sh-NC or sh-LINC00887 and further treated them with lentiviral sh-NC or sh-UPF1. LINC00887 knockdown notably curbed tumor growth in mice compared to controls; the sh-UPF1 treatment overcame the tumor-inhibiting effect initially induced by LINC00887 knockdown (**Figures 9A–C**). Through qRT-PCR data, we found that the sh-LINC00887+sh-NC treated group exhibited a marked reduction in LINC00887 expression versus the control group, accompanied by an upward trend in UPF1 mRNA level; sh-UPF1 treatment did not greatly alter LINC00887 expression but reduced UPF1 levels substantially (**Figures 9D, E**). Furthermore, IHC analysis showed that the sh-UPF1 lentiviral treatment counteracted the increase in UPF1 protein and the decrease in LDH-A and KI67 proteins caused by LINC00887 knockdown (**Figure 9F**). Additionally, the lactate levels in mouse tumor tissues were reduced following LINC00887 knockdown but increased with sh-UPF1 treatment (**Figure 9G**). These findings suggest that the LINC00887/UPF1 axis influences aerobic glycolysis and lactic acid accumulation in ccRCC. We analyzed the levels of M2 macrophages in tumor tissues using flow cytometry and immunofluorescence assays. Downregulation of LINC00887 reduced the proportion and infiltration of M2 macrophages in tumors, effects that were reversed by sh-UPF1 lentiviral treatment (**Figures 9H, I**). Downregulation of either UPF1 or LINC00887 reduced or raised CD8^+^ T cell proportion in tumors, respectively (**Figure 9J**). This suggests that LINC00887, through its regulation of UPF1, may promote aerobic glycolysis and lactate accumulation in ccRCC, inducing M2 macrophage polarization and suppressing CD8^+^ T cell antitumor activity, potentially contributing to aggressive progression and immune evasion of ccRCC.

**Figure 9:**
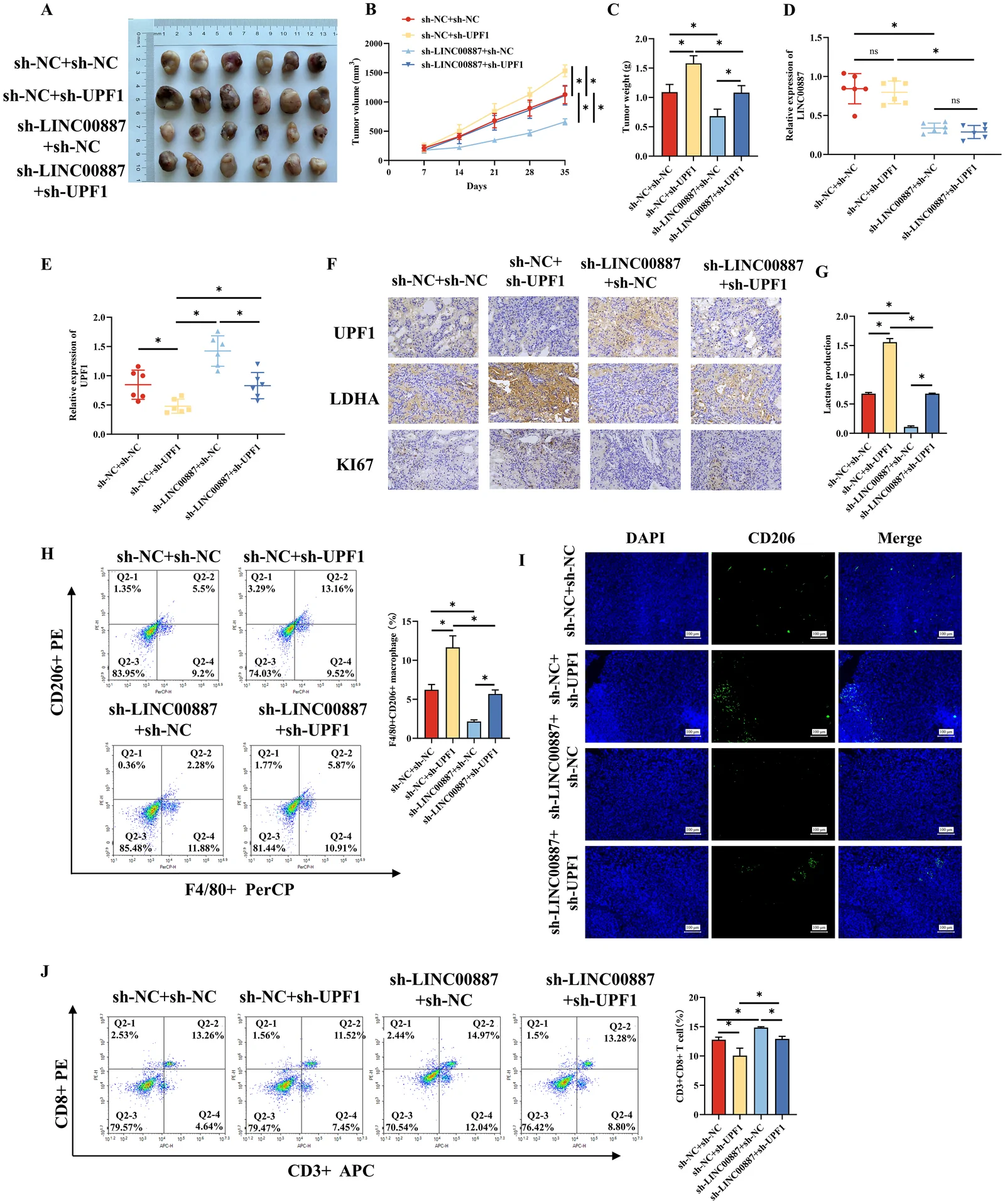
LINC00887/UPF1-mediated aerobic glycolysis leads to lactic acid accumulation and facilitates immune evasion in ccRCC *in vivo*, A syngeneic tumor model was developed by injecting BALB/c mice with Renca cells expressing sh-NC or sh-LINC00887, followed by treatment with sh-NC or sh-UPF1 lentivirus. A-C: Assessment of tumor size **(A)**, volume **(B)**, and weight **(C)**. **(D, E)** qRT-PCR analyses for LINC00887 **(D)** and UPF1 **(E)** mRNA levels. **(F)** IHC analysis for UPF1, LDH-A, KI67 protein levels. **(G)** Kit assay for lactate levels in tumors. **(H)** Proportion of M2 macrophages (F4/80+CD206+) by flow cytometry. **(I)** Immunofluorescence assay for CD206+ macrophages. **(J)** Proportion of CD8^+^ T cells (CD3^+^CD8^+^) in tumor tissues. ns, no significant difference. *p < 0.05. cRCC, Clear cell renal cell carcinoma; UPF1, Up-frameshift protein 1; LDH-A, Lactate dehydrogenase A; CD206, Mannose receptor C-type 1; CD3, Cluster of differentiation 3; CD8, Cluster of differentiation 8.

## 4 Discussion

As high-throughput sequencing and bioinformatics advance, a myriad of lncRNAs have been identified to exhibit differential expression in various cancers, with profound effects on tumor initiation and progression (26). LINC00887 is implicated in oncogenic functions in cancers, such as gliomas, nasopharyngeal carcinomas, and thyroid cancers (27–29). In this study, we identified a significant upregulation of LINC00887 in ccRCC tissues compared to both adjacent non-tumorous tissues and normal kidney cells, implicating its involvement in ccRCC pathogenesis. Elevated expression of LINC00887 also correlated with increased glycolysis in ccRCC cells, proposing that it could be essential in maintaining tumor growth and energy demands via the reprogramming of aerobic glycolysis. In a parallel development, a recent study has shown that LINC00887-encoded micropeptide ACLY-BP supports lipid accumulation in ccRCC by epigenetically stabilizing the expression of lipid metabolism enzymes, which in turn supplies energy for cell proliferation (30). Together, these findings establish LINC00887 as a key regulator of metabolic reprogramming in ccRCC and provide novel evidence for its role in modulating aerobic glycolysis, a hallmark of cancer malignancy.

LncRNAs interact with RNA, DNA, and proteins, which allows them to manipulate gene expression by affecting chromatin structure, post-translational modifications, and RNA stability, operating on multiple levels (31). Bioinformatics analysis predicted UPF1 as a potential target of LINC00887, with a significant negative correlation between their expression levels. Experimental validation demonstrated that UPF1 mRNA and protein levels were significantly increased following LINC00887 knockdown. Furthermore, we confirmed a direct interaction between LINC00887 and UPF1 protein. Previous studies revealed that LINC00887 suppresses SIRT3 levels and enhances H3K27 acetylation at the ETS1 promoter region, thereby manipulating downstream gene expression (32). Our results further confirmed that the increase in UPF1 protein levels induced by LINC00887 knockdown was not significantly affected by MG132. This indicates that LINC00887 primarily negatively regulates UPF1 expression at the transcriptional level. Based on the above findings, we hypothesized that in ccRCC, LINC00887 may suppress UPF1 transcription through a comparable histone modification-mediated transcriptional regulatory mechanism.

UPF1, recognized for its role in mRNA decay (33), is implicated in malignant progression of varying cancers (34), taking on different roles within these diseases (35). It can promote stemness in endometrial cancer and glioblastoma by stabilizing lncRNAs or circRNAs (36, 37), while in hepatocellular carcinoma, it acts as a suppressor of tumorigenesis (38). However, the role of UPF1 in ccRCC is yet to be determined. Our study demonstrated that LINC00887 functionally targetd and suppressed UPF1 expression in ccRCC. Single-cell RNA sequencing analysis revealed that UPF1 expression in malignant epithelial cells was decreased compared to their para-cancerous counterparts, reinforcing its tumor-specific regulatory role. *In vitro* testing demonstrated that UPF1 effectively curbed the malignancy of ccRCC cells. These findings are corroborated by recent research demonstrating that lncRNA-SLERCC exerts tumor-suppressive effects in ccRCC by stabilizing UPF1 and consequently suppressing the oncogenic Wnt/β-catenin signaling pathway (39). Collectively, these findings demonstrate that UPF1 functions as a tumor suppressor in ccRCC, whereas LINC00887 may facilitate tumorigenesis at least partially by downregulating UPF1 expression. As a core effector of the NMD pathway, UPF1 binds to mRNAs in a 3’UTR length-dependent manner to mediate mRNA stabilization or degradation (40, 41). For instance, UPF1 stabilizes HK2 mRNA in pancreatic cancer (42), while it exerts tumor-suppressive effects by repressing glycolysis in hepatocellular carcinoma (43). These observations indicate that the regulatory role of UPF1 in glycolysis exhibits cancer type specificity. Given the above evidence, we hypothesize that UPF1 may directly target the mRNAs of key glycolytic enzymes including LDH-A, HK2 and GLUT1 in ccRCC. Further investigations involving RNA stability assays, combined with genome-wide target identification approaches such as RIP-seq or CLIP-seq, are required to directly validate the detailed mechanism underlying this regulatory axis in ccRCC.

In TME, there is a complex and dynamic relationship between tumor cells, macrophages, and other elements. M2 macrophages, the functional opposite of M1 macrophages, are key in fostering the growth and spread of cancer cells (44). An existing study demonstrates that risk models constructed based on M2 macrophage-related genes exhibit high predictive value for ccRCC patient prognosis (45). Furthermore, research has confirmed that RCC-derived exosomes promote tumor progression by inducing macrophage M2 polarization (45). These findings collectively indicate that M2 macrophage infiltration in TME drives ccRCC and represents a key factor in ccRCC treatment assessment. The aberrant aerobic glycolysis in tumor cells transforms glucose into lactate, and the overproduction of lactate acidifies the TME, being closely related to M2 macrophage polarization (46). Our investigation delineates an essential oncogenic pathway in ccRCC: elevated LINC00887 expression increases lactate generation and induces tumor-associated macrophage polarization to the protumor M2 subtype. Importantly, through integrated scRNA-seq analysis and functional validation, we identify UPF1 as a central regulator mediating these metabolic reprogramming events in ccRCC glycolysis. *In vivo* experiments further validate that LINC00887 enhances M2 macrophage infiltration and depletes CD8^+^ T cells in ccRCC by downregulating UPF1. While it is known that downregulating UPF1 can stimulate glycolysis in liver cancer (43), the function of UPF1 in pancreatic cancer is quite different, where it enhances glycolytic flux by stabilizing HK2 and helps to maintain an immunosuppressive tumor microenvironment (42). This finding demonstrates the functional heterogeneity of UPF1 in regulating aerobic glycolysis across different cancer types.

We identified significant overexpression of LINC00887 in ccRCC tissues in comparison to normal tissues. Experiments showed that LINC00887 targeted UPF1, which reprogrammed the metabolic pathways of ccRCC cells and led to lactate buildup. This buildup was associated with macrophage polarization to M2 and supported the immune evasion capabilities of the tumor. The LINC00887/UPF1 axis was found to be a key regulator of energy metabolism that shapes the immunosuppressive tumor microenvironment in ccRCC. Thus, targeting LINC00887 might offer a boost to the effectiveness of immunotherapies in ccRCC, opening new therapeutic possibilities. Nevertheless, several limitations of this study should be acknowledged. First, the single-cell RNA-sequencing analysis was performed based on only two pairs of tumor and adjacent normal tissue specimens, which compromises its statistical power. Second, the correlation between LINC00887 and UPF1 expression remains to be validated via immunohistochemical detection in clinical samples. Third, the precise molecular mechanism by which LINC00887 inhibits UPF1 expression, as well as the direct RNA targets of UPF1 in ccRCC, still require further clarification through RNA stability analysis, RIP-seq/CLIP-seq, and domain mapping assays. Furthermore, only pharmacological inhibitors (OXM and 2-DG) were adopted in the present study to confirm the regulatory role of lactic acid in macrophage polarization. Finally, although the MG132 experiment has ruled out a major contribution of the proteasomal pathway, whether the autophagy-lysosomal pathway is involved in LINC00887-mediated UPF1 protein degradation remains unclear. In subsequent research, we will further investigate the potential role of the autophagy pathway. Rescue experiments will be conducted by supplementing exogenous sodium lactate into the culture medium of LINC00887-knockout or UPF1-overexpressed cells, to further elaborate the functional role of lactic acid in M2 macrophage polarization.

## Data Availability

The datasets generated and analysed during the current study are available in the GEO repository, Accession number: GSE301681.

## Supplementary material

Supplementary Figure 1 Expression levels of LINC00887 in ccRCC with different pathological characteristics. **(A–D)** Violin plots showing the expression distribution of LINC00887 in ISUP grade **(A)**, T stage **(B)**, N stage **(C)**, and pathological stage **(D)** based on the TCGA-KIRC dataset. **p* < 0.05, ***p* < 0.01, ****p* < 0.001, *****p* < 0.0001. cRCC, Clear cell renal cell carcinoma; KIRC, Kidney renal clear cell carcinoma.

Supplementary Figure 2 LINC00887 promotes aerobic glycolysis in Caki-1 cells. Caki-1 cells were transfected with sh-NC or sh-LINC00887, respectively. **(A)** qRT-PCR was performed to verify transfection efficiency. **(B)** qRT-PCR measurement of glycolytic marker genes LDH-A, HK2, and GLUT1 mRNA levels in each group. **(C)** immunoblotting analysis of glycolytic marker LDH-A, HK2, and GLUT1 protein levels in each group. **(D)** Seahorse metabolic analyzer was used to measure ECAR in each group, calculating glycolytic value and glycolytic capacity. **(E)** Seahorse metabolic analyzer was used to measure OCR in each group, calculating ATP production, and maximal respiration. **(F–H)** Kits were used to measure glucose consumption **(F)**, lactate production **(G)**, and ATP yield **(H)**. * indicates *p* < 0.05. qRT-PCR, Quantitative reverse transcription polymerase chain reaction; LDH-A, Lactate dehydrogenase A; HK2, Hexokinase 2; GLUT1, Glucose transporter 1; ECAR, Extracellular acidification rate; OCR, Oxygen consumption rate; ATP, Adenosine triphosphate.

Supplementary Figure 3 LINC00887 enhances lactic acid accumulation through aerobic glycolysis to drive M2 polarization in ccRCC, **(A)** Pearson correlation analysis between LINC00887 and immune cell infiltration in ccRCC. **(B)** Pearson correlation analysis of LINC00887 with M2 macrophages calculated using the CIBERSORT-ABS algorithm. M0 macrophages were cultured with supernatants from 786-O cells treated with oe-NC+PBS, oe-LINC00887+PBS, or oe-LINC00887+OXM. **(C, D)** qRT-PCR analysis of mRNA levels for M1 markers (TNF-α, iNOS, IL-1β) and M2 markers (Arg1, Fizz1, IL-10) in the different groups. **(E, F)** Flow cytometry analysis of M1 (CD68^+^CD86^+^) and M2 (CD68^+^CD206^+^) macrophage counts. * indicates *p* < 0.05. cRCC, Clear cell renal cell carcinoma; OXM, Oxamate; TNF-α, Tumor necrosis factor-alpha; iNOS, Inducible nitric oxide synthase; IL-1β, Interleukin - 1β; Arg1, Arginase 1; Fizz1, Found in inflammatory zone 1; IL-10, Interleukin -10; CD68, Cluster of differentiation 68; CD86, Cluster of differentiation 86; CD206, Mannose receptor C-type 1.

Supplementary Figure 4 Distribution and metabolic impact of UPF1 in ccRCC, **(A)** KEGG pathway analysis comparing ccRCC tumor and adjacent normal tissues. **(B)** KEGG pathway analysis across the seven predominant cell subtypes. **(C)** Umap representation of UPF1 expression in ccRCC tumor versus adjacent normal tissues across all cell types. **(D)** Umap visualization following reclustering of epithelial cells. **(E, F)** Umap plots indicating epithelial cells with the marker genes EPCAM **(E)** and KRT18 **(F)**. **(G)** Umap visualization of UPF1 expression in epithelial cells within ccRCC tumor and adjacent normal tissues. **(H)** KEGG analysis depicting metabolic pathways enriched by UPF1. **(I)** Network diagram of cell-cell communication quantity. Left: Paracancerous tissue; Right: Tumor tissue. Node size represents cell number, and connection thickness indicates the number of communication pairs. UPF1, Up-frameshift protein 1; cRCC, Clear cell renal cell carcinoma; KEGG, Kyoto Encyclopedia of Genes and Genomes; EPCAM, Epithelial cell adhesion molecule; KRT18, Keratin 18.

Supplementary Figure 5 Expression levels of UPF1 in ccRCC with different pathological characteristics. **(A–D)** Violin plots showing the expression distribution of UPF1 in ISUP grade **(A)**, T stage **(B)**, N stage **(C)**, and pathological stage **(D)** based on the TCGA-KIRC dataset. **(E, F)** Kaplan-Meier survival curves of overall survival (OS) **(E)** and progression-free survival (PFS) **(F)** based on the TCGA-KIRC dataset. **p* < 0.05, ***p* < 0.01, ****p* < 0.001, *****p* < 0.0001. cRCC, Clear cell renal cell carcinoma; KIRC, Kidney renal clear cell carcinoma.

Supplementary Figure 6 LINC00887 regulates aerobic glycolysis by interacting with UPF1 in ccRCC, Caki-1 cells were transfected with sh-NC + sh-NC, sh-LINC00887 + sh-NC, sh-NC + sh-NC + sh-UPF1, or sh-LINC00887 + sh-UPF1, **(A)** qRT-PCR analysis of mRNA levels of UPF1, LDH-A, HK2, and GLUT1 in each group. **(B)** Immunoblotting analysis of protein levels of UPF1, LDH-A, HK2, and GLUT1 in each group. 786-O cell groups of sh-NC + sh-NC, sh-LINC00887 + sh-NC, or sh-LINC00887 + sh-UPF1, **(C)** ECAR measured by Seahorse metabolic analyzer, with calculated glycolysis, glycolytic capacity, and glycolytic reserve values. **(D)** OCR measured by Seahorse metabolic analyzer, with calculated basal respiration, ATP production, and maximal respiration values. Assay kits for glucose consumption **(E)**, lactate production **(F)**, and ATP production **(G)**. **p* < 0.05. UPF1, Up-frameshift protein 1; cRCC, Clear cell renal cell carcinoma; qRT-PCR, Quantitative reverse transcription polymerase chain reaction; LDH-A, Lactate dehydrogenase A; HK2, Hexokinase 2; GLUT1, Glucose transporter 1; ECAR, Extracellular acidification rate; OCR, Oxygen consumption rate; ATP, Adenosine triphosphate.

Supplementary Figure 7 UPF1 regulates aerobic glycolysis in ccRCC to inhibit macrophage M2 polarization, Caki-1 cell groups of oe-NC + oe-NC, oe-UPF1 + oe-NC, or oe-UPF1 + oe-GLUT1, **(A)** Immunoblotting analysis of protein levels of UPF1, LDH-A, HK2, and GLUT1 in each group. **(B)** ECAR measured by Seahorse analyzer (glycolytic capacity, glycolysis, glycolytic reserve). **(C)** OCR measured by Seahorse analyzer (ATP production, basal respiration, maximal respiration). **(D–F)** Assay kits for glucose consumption **(D)**, lactate production **(E)**, and ATP production **(F)**. Conditioned medium from differently treated Caki-1 cells was used to culture M0 macrophages, **(G, H)** Flow cytometry quantification of M1 macrophages (CD68^+^CD86^+^) and M2 macrophages (CD68^+^CD206^+^). **(I, J)** qRT-PCR analysis of M1 markers [TNF-α, iNOS, IL-1β; **(I)**] and M2 markers [Arg1, Fizz1, IL-10; **(J)**]. *, *p* < 0.05. UPF1, Up-frameshift protein 1; cRCC, Clear cell renal cell carcinoma; LDH-A, Lactate dehydrogenase A; HK2, Hexokinase 2; GLUT1, Glucose transporter 1; ECAR, Extracellular acidification rate; OCR, Oxygen consumption rate; ATP, Adenosine triphosphate; CD68, Cluster of differentiation 68; CD86, Cluster of differentiation 86; CD206, Mannose receptor C-type 1; TNF-α, Tumor necrosis factor-alpha; iNOS, Inducible nitric oxide synthase; IL-1β, Interleukin - 1β; Arg1, Arginase 1; Fizz1, Found in inflammatory zone 1; IL-10, Interleukin -10.
